# Pro-Tumorigenic Macrophage Infiltration in Oral Squamous Cell Carcinoma and Possible Macrophage-Aimed Therapeutic Interventions

**DOI:** 10.3389/fonc.2021.675664

**Published:** 2021-05-10

**Authors:** Flavia Bruna, Pablo Scodeller

**Affiliations:** ^1^ Consejo Nacional de Investigaciones Científicas y Tecnicas, Universidad Nacional de Cuyo, Instituto de Medicina y Biología Experimental de Cuyo, Mendoza, Argentina; ^2^ Institute of Biomedicine and Translational Medicine, University of Tartu, Tartu, Estonia

**Keywords:** oral squamous cell carcinoma, tumor-associated macrophage, CD206 receptor, targeting peptides, drug delivery, tumor microenvironment

## Abstract

In Oral Squamous Cell Carcinomas (OSCC), as in other solid tumors, stromal cells strongly support the spread and growth of the tumor. Macrophages in tumors (tumor-associated macrophages or “TAMs”), can swing between a pro-inflammatory and anti-tumorigenic (M1-like TAMs) state or an anti‐inflammatory and pro-tumorigenic (M2-like TAMs) profile depending on the tumor microenvironment cues. Numerous clinical and preclinical studies have demonstrated the importance of macrophages in the prognosis of patients with different types of cancer. Here, our aim was to review the role of M2-like TAMs in the prognosis of patients with OSCC and provide a state of the art on strategies for depleting or reprogramming M2-like TAMs as a possible therapeutic solution for OSCC. The Clinical studies reviewed showed that higher density of CD163+ M2-like TAMs associated with worse survival and that CD206+ M2-TAMs are involved in OSCC progression through epidermal growth factor (EGF) secretion, underlining the important role of CD206 as a marker of OSCC progression and as a therapeutic target. Here, we provide the reader with the current tools, in preclinical and clinical stage, for depleting M2-like TAMs, re-educating them towards M1-like TAMs, and exploiting TAMs as drug delivery vectors.

## Oral Squamous Cell Carcinoma

Oral squamous cell carcinoma (OSCC) that does not originate from papilloma virus is a solid tumor of epithelial oral cells (1). The World Health Organization reported that the mortality rate in OSCC has remained largely unchanged for the last decades, with the 5-year survival being of 50% (Institute NC: Surveillance, Epidemiology, and End Results Program of the National Cancer Institute. In. https://seer.cancer.gov.; 2016). The high mortality rate and rate of recurrence in OSCC are suggested to be related in part to the loco-regional invasion capacity and metastasis to cervical lymph nodes (2, 3). The tumor microenvironment or reactive stroma, comprised of extracellular matrix, fibroblasts, myofibroblasts, endothelial cells, adipocytes, and immune effector cells, influences tumor progression and metastasis (4, 5). Events related to the oral epithelial cancer pathogenesis take place in a dysregulated microenvironment, affecting both normal and neoplastic cells (6–9). The disruption of tissue homeostasis contributes to OSCC growth through interactions between the tumor cells, a reactive stroma and mediators of the immune system including macrophages (10–12). The current consensus is that during carcinogenesis, tumor progression depends on the cross-talk between tumor cells, stromal cells, and the host inflammatory cells. Although the effects of tumor cell/stroma interaction on oncogenesis in OSCC have been studied, those studies focused on the cellular parameters involved in transforming epithelial cells (8, 9) and the pro-tumorigenic potential of the local immune infiltrate during the OSCC progression was not taken into account.

In recent years, the interest in the role of innate and adaptive immune cells in carcinogenesis has surged greatly. Several studies, in both animal models and human biopsies, have shown that chronic inflammation increases the risk of cancer appearance (13–15), but once a tumor has established, anti-inflammatory, or M2-like tumor associated macrophages, (M2-like TAMs) strongly contribute to support its growth (16).

## Macrophage Differentiation

In adults, inflammatory monocytes give origin to tissue-resident macrophage populations (17, 18). Macrophages are involved in various essential aspects of host defense mechanisms and pathophysiological conditions, such as chronic inflammatory disease and cancer (19, 20). Previous studies reported that in biopsies of lung adenocarcinoma patients, the macrophage phenotype depends on the exposure to numerous stimuli present in the tissue microenvironment (19), which results in a complex phenotypic population in a time and location-dependent manner (17, 21). The activation *in vitro* of different regulatory mechanisms and transcription pathways results in a vast spectrum of macrophage subtypes, of which the states referred to as M1 and M2 represent the extreme polarization phenotypes (21, 22). The M1 polarization state depends on microbial stimulus and a T helper type 1 (Th1) cytokine profile. Bacterial cellular components, such as lipopolysaccharide (LPS), and the Th1-derived cytokine interferon-gamma (IFN-*γ*) polarize towards the so called “classically activated” macrophages, referred to as M1 macrophages. These macrophages produce pro-inflammatory cytokines, such as IL-12 and tumor necrosis factor-alpha (TNF-α), reactive oxygen intermediates, and reactive nitrogen intermediates, giving them anti-microbial and anti-tumoral activities (23, 24). In contrast, M2 polarization depends on a T helper type 2 (Th2) cytokine profile (25), and Th2-derived IL-4 and IL-13. M2 macrophages (also called “alternatively activated”) participate in anti-inflammatory processes, tissue remodeling, and angiogenesis (23, 24). Interferon-gamma (IFN-*γ*) and interleukin (IL)-4 secretion sustain an M1 and an M2 phenotype commitment, respectively (26). The expression of some markers or secreted factors has shown to be different for mouse than for human macrophages. For instance, Martinez et al. observed that IL-4 and IL-13 do not induce the human homolog of the mouse M2 markers arginase 1, Fizz1, MMP1 and Ym1 (27).

M2 macrophages differentiate in the tumor stroma from blood monocytes, or resident macrophages in resting state, when exposed to the aforementioned cytokines aberrantly expressed by neoplastic cells (28). M2 macrophages block Th1 cells, and recent studies of OSCC patients’ biopsies have confirmed that tumor-associated macrophages (TAMs) involved in tumor progression display an M2 phenotype and contribute to tumor angiogenesis, invasion, and metastasis also in OSCC (23, 29).

Several studies from biopsies of human malignant tumors have described that M2 TAMs highly express the markers CD163, CD204, and CD206 (30). CD204 belongs to a family of transmembrane receptors known as scavenger receptors, which are primarily expressed on macrophages and dendritic cells (30). CD204 recognizes modified lipoproteins, and exogenous pathogen-associated molecular patterns, and apoptotic cells (30). CD163 is a member of the scavenger receptor cysteine-rich family and is mainly expressed on mature tissue macrophages. One of the functions of CD163 is to internalize the hemoglobin–haptoglobin complex, involved in the resolution of the inflammation (31). CD163^+^/CD204^+^ TAMs have been reported to promote T-cell apoptosis and immunosuppression *via* IL-10 and programmed death-ligand 1 (PD-L1) in OSCC patients (32). CD206 is a C-type lectin, also known as the macrophage mannose receptor, expressed on tissue macrophages, dendritic cells, and to a lesser extent on some lymphatic vessels (33) and on sinusoidal endothelial liver cells (34, 35). CD206 plays an important role in immune homeostasis and contributes to lipid metabolism, atherogenesis, and metabolic processes (29), but it is aberrantly expressed on macrophages in the tumor microenvironment (36). CD206^+^ M2 TAMs promote cancer progression by STAT-3 activation, inducing and maintaining a pro-carcinogenic microenvironment secreting high levels of VEGF, TGF-β, EGF, uPA, and several matrix metalloproteases (MMPs) promoting tumor progression, immunosuppression, angiogenesis, migration, metastasis and chemoresistance (37, 38). These pro-tumoral TAMs also secrete low amounts of IL-12 and have impaired nitric oxide induction.

**Figure 1:**
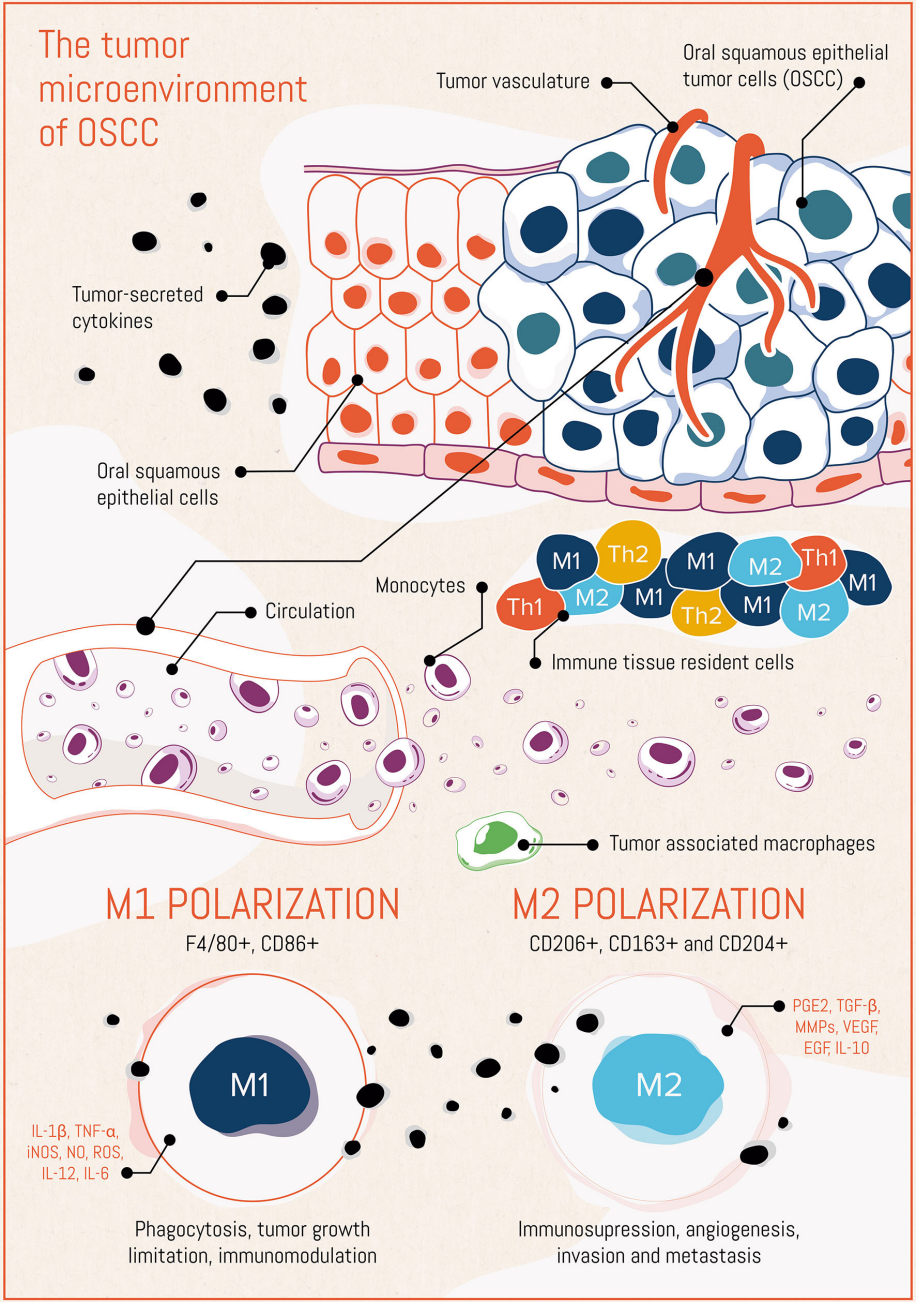
The tumor microenvironment in oral squamous cell carcinoma (OSCC), placing emphasis on the macrophage compartment.

The phenomena and mechanisms described in this section are schematically summarized in **Figure 1**.

## OSCC Cells and M2-Like TAMs Interactions

M2-like TAMs are the primary component of anti-inflammatory cells in the microenvironment of many solid tumors, including OSCC (22, 39). M2-like TAMs can induce the progression and metastatic spread of OSCC, promoting angiogenesis, tumor cell invasion, cell motility, persistent growth, and suppression of anti-tumor responses (40, 41). In turn, the tumor cells influence macrophage physiology to display a pro-tumor phenotype of TAMs to favor OSCC progression (28).

An increase in the number of M2-like TAMs was shown to occur during the progression of OSCC (17) and was associated with angiogenesis and higher histopathological grades in human tumor biopsies (18, 42).

Histopathologically, OSCC presents fibrous connective tissue with unusual amounts of extracellular matrix, rich in fibroblasts, blood vessels, and inflammatory cells (43). Among the local milieu, macrophages are differentiated into a diverse TAM population with varying expression of CD68, CD163, CD204, and CD206. These cells present biological importance for disease progression. Their number is correlated with a lower degree of differentiation in primary tumor sites and poor disease prognosis (15, 44). Moreover, M2-like TAMs elicit tumor relapse and/or postoperative cervical lymph node metastasis *via* angiogenesis and suppression of anti-tumor immunity (41). An increase in the number of CD163+ macrophages occurs in potentially malignant oral lesions such as leukoplakia (45). It has been suggested that in premalignant lesions TAMs are more skewed towards the M1 phenotype (42). The polarization to M2-like TAM phenotype probably occurs gradually and early during the onset of OSCC and is sustained by several interleukins (IL-1, -4, -6, -8, and -10), and other factors, such as the receptor tyrosine kinase Axl (28). Hence, the presence of M1-like and M2-like TAMs could be used as a potential marker to distinguish incipient OSCC from invasive lesions, avoiding under diagnoses (46). The healthy oral mucosa lacks a standard structure making the detection of invasiveness in oral cancer challenging. However, from the available evidence, it is possible to suggest that screening for TAM markers in oral biopsies certainly may contribute to accurate assessment of OSCC behavior, being a valuable tool for the estimation of prognosis in cases related and unrelated to viral infection (47, 48). Weber et al. proposed that even trauma from incisional biopsies might influence tumor biology leading to a worse prognosis and increased risk of developing lymph node metastases in OSCC patients (44). A wound-healing reaction consecutive to tissue trauma might provide a microenvironmental stimulus that affects macrophage polarization (49).

The importance in malignancy of M2-like TAMs is a general feature in many solid tumors besides OSCC; several studies indicated that CD163^+^, CD204^+^, and CD206^+^ M2-like macrophages that infiltrate the tumor microenvironment are significantly associated with poor prognosis in patients with lung cancer (50, 51), hepatocellular carcinoma, breast cancer, cervical cancer, multiple myeloma, and B-cell lymphoma (52).

Soluble CD163 was found to be a potential diagnostic parameter for monitoring the presence of macrophages, both in human biopsies of non-small cell lung cancer (NSCLC) (50) and human esophageal squamous cell carcinoma line (51). The mannose receptor CD206 is strongly expressed in prostate adenocarcinoma biopsies, and the number of CD206^+^ M2-like TAMs correlated with poor prognosis of the disease (53). Several reports describe the link between CD163^+^ M2-like TAMs and OSCC stages III–IV and implicate them in the poor prognosis (41, 54).

Haque et al. (53) recently demonstrated that CD206^+^ M2-like TAMs better predict unfavorable clinical prognosis in OSCC patients than CD163^+^ or CD204^+^ M2-like TAMs. They performed a series of elegant experiments using different M2-like subsets (CD163^+^, CD204^+^ or CD206^+^) isolated from peripheral blood mononuclear cells (PBMCs) from OSCC patients. Flow cytometry on these cells revealed that the number of EGF^+^ cells better correlated with the expression of CD206 than with the expression of CD163 or CD204, and ELISA assays determined that the CD206^+^ subset produced much higher levels of EGF than the CD163^+^ or CD204^+^ subset. OSCC cells (from an OSCC cell line) incubated with different M2-like subsets (differentiated and sorted from healthy human PBMCs), revealed that the CD206^+^ subset induced a higher production of EGF (determined using ELISA) and a significantly higher proliferation and invasion of the OSCC cells, than the CD163^+^ or CD204^+^ subset. Based on these experiments, they proposed that the higher EGF secretion generated by the CD206^+^ subset likely accounts for the correlation of this subset with poor clinical prognosis.

Therefore, it is important to determine the phenotype of TAMs in each type of tumor to understand the process of tumor progression and to evaluate the prognosis of the patients (48). The features of different studies of TAM characterization in OSCC are summarized in **Table 1**.

**Table 1:** Main human studies of TAMs in OSCC.

References	Numberof patients	Type of cancer and TNM staging (T1, T2)	Median patient age (range)	TAM markers assessed	Methodology Immunohistochemistry (IHC)	TAMs phenotype/OSCC grade	Conclusions
Wehrhan et al. (41)	37	OSCC (T1, T2)	Not reported	CD68 CD11c CD163 CD206	IHC	M2-like/high grade OSCC	CD206^+^ M2-like TAMs influenced lymph node metastasis formation in OSCC.
Fujii et al. (54)	108	OSCC (not reported)	66.4 (23–93)	CD68 CD163	IHC	M2-like/high grade OSCC	Cancer associated fibroblasts (CAFs), promoted an immunosuppressive microenvironment, inducing M2-like phenotype.
Mori et al. (29)	50	OSCC (T1, T2 and T3)	50 (35–80)	CD163 CD80 CD68	IHC	M2-like/high grade OSCC	Infiltrating M2-like TAMs participated in OSCC development.
Haque et al. (53)	65	OSCC (T1, T2 and T3)	66.5 (35–89)	CD163, CD204, CD206	IHC	M2-like/high grade OSCC	CD206^+^ M2-like TAMs correlated with worse clinical prognosis of OSCC.
Wang et al. (48)	298	OSCC (not reported)	50 (35–80)	CD163, CD80, CD68	IHC	M2-like/high grade OSCC	Cancer-associated fibroblasts and CD163^+^ M2-like TAMs correlated with the clinical prognosis of OSCC.

## Ligands Targeting M2-Like TAMs and Potential Therapeutic Strategies for OSCC

Due to the multiple malicious actions that M2-like TAMs take in the progression of OSCC disease, it becomes important, from a translational point of view, to be able to target M2-like while sparing M1-like TAMs. Possible macrophage-aimed interventions include the depletion of M2-like TAMs using apoptotic agents or the conversion of M2-like to the M1-like phenotype (55). Although TAMs have been detected in oral squamous cell carcinoma (OSCC), little is known on how therapeutically targeting them affects the disease.

Studies in mice (56) and humans (57) that deplete tumor macrophages to potentiate checkpoint inhibitor immunotherapy (CPI), target receptors (CSF1R, CCR2, CD40) that do not precisely identify the M2-like subset of macrophages. These receptors are also expressed on tumoricidal M1-like TAMs and microglia (58–60). Most likely, targeting those receptors is not the most effective strategy to potentiate CPI, as it will deplete M1-like TAMs, and also result in side effects (61). More recently, another protein, TREM2, has been identified as a marker of immunosuppressive tumor-associated macrophages (62). The company Pionyr Immunotherapeutics has developed an antibody against TREM2, called PY314, that depletes TREM2+ TAMs by antibody dependent cell mediated cytotoxicity (ADCC) which in turn reduces the amount of exhausted CD8+ T cells. The company has recently received FDA approval to start Phase I clinical trials of PY314 in cancer patients.

Antibodies against CD163 have been used to target M2-like TAMs in mouse models of melanoma (63). In that work, *α*-CD163 was coated on the surface of liposomes encapsulating doxorubicin, to deplete M2-like TAMs and potentiate CPI. On the other hand, targeting the mannose receptor (CD206) represents an appealing avenue, as CD206 identifies M2-like TAMs and precursors of metastasis-associated macrophages (64), and as we exposed in the previous sections, the number of CD206^+^ M2-like TAMs correlates with malignancy and poor prognosis in OSCC.

However, because of the high stromal content of tumors (including OSCC), the high molecular weight of antibodies, and the phenomenon known as the binding site barrier (wherein a very high affinity precludes penetration) (65), the diffusion of antibodies in a tumor is seriously hampered (66, 67). An alternative is to use nanobodies, which possess much lower molecular weights (approx. 15 kDa). Ginderachter et al. have developed a CD206-targeting nanobody that was efficient in targeting M2-like TAMs in mouse models of lung and breast carcinoma (68). However, this nanobody also presented accumulation in the liver, perhaps stemming from its high affinity for CD206, which is also expressed in the liver. This *α*-CD206 nanobody, conjugated to a 68Ga-chelating moiety, is now in phase I/IIa as a diagnostic contrast agent for PET imaging in solid cancers (ClinicalTrials.gov Identifier: NCT04168528).

The company Navidea developed mannose-based ligands to target the mannose-binding site of CD206 (69) (Manocept™, clinically used in the contrast agent Lymphoseek^®^). However, those ligands are not specific to CD206 because other mannose binding proteins exist in the body [like CD209 in intestinal and genital tissues (70)]; hence Lymphoseek^®^ has to be administered locally. In a preclinical study, Zhang et al. (71) used mannose coating to guide therapeutic nanoparticles to CD206+ M2-like TAMs. In that work, mannose-coated polymeric nanoparticles containing mRNAs encoding interferon regulatory factor 5 (IRF5) and its activating kinase IKK*β*, administered systemically, reverted M2-like TAMs to M1-like TAMs and increased the survival of mice with ovarian cancer, experimental metastasis of melanoma, and glioma, without any observed systemic toxicity. Another company, Riptide Inc., developed a peptide called RP-182 that binds to CD206. This peptide was recently reported to induce an M2-like➔M1-like switch in tumor macrophages, mediated by ligand-induced conformational changes on CD206 (72). RP-182 was also shown to bind to RelB, Sirp-*α* and CD47 (73), raising questions on its M2-like TAMs exclusiveness.

Targeting peptides are appealing tumor-guiding agents for nanoparticles (74–79) and also for lower molecular weight therapeutic and imaging agents (72, 80–82). These targeting peptides, most of which have been identified using *in vivo* phage display (80), have high selectivity for their targets and high tumor penetration. Additionally, they can be chemically modified to improve certain properties such as affinity, stability to proteases, and oral availability (80).

We identified and published a CD206-targeting peptide, called “mUNO”, that binds to mouse and human CD206 (83), is selective to mouse and human CD206+ macrophages (84), and delivers payload specifically to M2-like TAMs in mouse models of melanoma, glioblastoma, gastric carcinoma, and breast cancer (83) with very low accumulation in healthy organs (83, 85). We showed that mUNO binds to a different binding site than mannose on CD206 (84), making it more specific to CD206 than mannose-based ligands. Additionally, mUNO only targets CD206 in the tumor and not in the liver; a moderate affinity of mUNO for CD206, and a higher dwelling time in tumor than in liver (due to leaky tumor vasculature) may explain this phenomenon (85). In a recent publication (86), we showed that the drug resiquimod was able to enhance chemotherapy in triple negative breast cancer in mice when it was loaded in nanoparticles targeted to M2-like TAMs using mUNO; while the untargeted, resiquimod-loaded nanoparticles showed no effect on potentiating chemotherapy. Resiquimod is a known agonist of the toll like receptors TRL7/8 and in macrophages reverses the M2-like phenotype into an M1-like phenotype. However, un-targeted TLR7/8 agonists, including resiquimod, cannot be delivered systemically because it leads to high levels of systemic IFN-*γ* and toxic side effects (87, 88). In our work, resiquimod encapsulated in mUNO-coated lignin nanoparticles slowed down the tumor growth and significantly transformed the immune landscape in the tumor; decreased M2-like TAMs, and increased CD8 T-cells, M1-like TAMs, activated dendritic cells and IFN-*γ*. We did not observe these effects with free resiquimod or untargeted, resiquimod-loaded, nanoparticles. In cancer nanotherapeutics designed to attack M2-like TAMs, the use of active M2-like targeting is expected to be superior, because, as all macrophages have nanoparticle-phagocyting activity, the uptake by M1-like TAMs will result in decreased efficiency (for the case of reprogramming nanotherapeutics), or undesired depletion of M1-like TAMs (for the case of depleting nanotherapeutics).

Folate (Mw: 441 Da) has also been used to successfully target M2-like TAMs, as the folate receptor-β has been shown to be over-expressed on M2-like TAMs (89). The group of Dr. Philip Low has recently used a folate-drug conjugate to repolarize M2-like macrophages to M1-like macrophages in pulmonary fibrosis (M2-like macrophages secrete fibrosis-inducing cytokines) which led to a reduction of stromal content (90) and beneficial therapeutic effect in their mouse model of pulmonary fibrosis. Even if this study was conducted in pulmonary fibrosis, it could be applied to switch M2-like TAMs to M1-like TAMs. Folate as a targeting ligand for cancer is currently in phase III clinical trials (NCT03180307).

The features of different targeting ligands are summarized in **Table 2**.

**Table 2:** Ligands targeting M2-like macrophages.

Ligand	Affinity	Penetration	Clinical stage	Examples (Receptor). Reference
**Peptides** (4–12mer, linear or disulfide-cyclized)	Low-medium (low µM range)	High	Preclinical	RP-182 (Receptor: CD206) (72) mUNO (Receptor: CD206) (85) M2Pep (Receptor: unidentified) (82)
**Antibodies**	High (low nM range)	Low	Preclinical or Phase I/II (*α*-TREM2, *α*-CSF1R)	*α*-CSF1R (57) *α*-TREM2 (62) *α*-CD163 (63)
**Nanobodies**	High (high nM range)	Intermediate	Phase I/IIa	CD206-targeting nanobody (Receptor: CD206) (68)
**Small molecule ligands**	Medium	High	Approved (mannose-based Lymphoseek) Or in Phase III (Folate)	Mannose (Receptor: CD206) (69) Folate (Receptor: Folate receptor-*β* (90)

## Macrophages as Drug Delivery Vehicles

Macrophages have an inherent ability to navigate desmoplasia-dense tumor regions, a feature that in M2-like macrophages is partly mediated by their high matrix metallopeptidase 9 (MMP-9) activity (91, 92). Because of their capacity to penetrate the extravascular space, to migrate to and reside in the tumor, several studies have used TAMs as transportation vehicles for pharmacological agents.

Miller et al. (93) showed that TAMs slowly released a platinum pro-drug encapsulated in polymeric nanoparticles, acting as slow release drug depots in the tumor. The drug-loaded nanoparticles were composed of (poly(lactic-co-glycolic acid)-poly(ethylene glycol) (PLGA-PEG) and were administered intravenously in mice with subcutaneous fibrosarcoma tumors. However, this study did not dissect whether it was M1-like TAMs or M2-like TAMs, or both, that displayed the observed drug reservoir effect. In a recent study (86), we observed that M2-like TAMs and not M1-like TAMs took up untargeted lignin nanoparticles; but there was a significantly higher accumulation of nanoparticles/M2-like TAM when they were coated with a CD206-binding peptide.

In a recent publication, Wyatt Schields et al. (94) designed discoidal, micrometer sized (7 µm-wide), particles containing IFN-*γ* and a cell-adherent layer constructed using layer by layer self-assembly of hyaluronic acid and poly(allylamine). They then used an adoptive cell transfer approach, wherein bone marrow-derived monocytes were incubated with the backpacks (stimulating them to become M1-like macrophages) and were later injected intratumorally in breast tumors in mice. The introduced, backpack-carrying, M1-like macrophages released IFN-*γ* in the tumor and skewed M2-like TAMs to M1-like TAMs. This treatment led to reduced metastasis and slower tumor growth compared to mice that received the same dose of macrophages plus free IFN-*γ*, which is explained by a favorable release kinetics of the backpack-encapsulated IFN-*γ*. According to the authors, the anisotropy of the particles was responsible for the evasion of macrophage phagocytosis (95).

Torrieri et al. (96) used a p32 (gC1qR/HABP1)-targeting peptide (named “linTT1”, sequence: AKRGARSTA) to direct nanoparticles to M2-like macrophages and utilized them to hitch-hike their cargo-loaded nanoparticles to infarcted heart tissue, taking advantage of the post-infarction recruitment of M2-like macrophages. LinTT1 targets p32, a protein which is mainly localized in the mitochondria but has also been shown to be expressed on the surface of tumor macrophages (97, 98), hence in principle, the same approach used by Torrieri et al. could be applied to M2-like TAMs for cancer therapy.

Du Nguyen et al. (99) used macrophages from the cell line RAW 264.7, as drug carriers or “trojan horses” for multiple nanotherapeutics incorporated ex vivo. Therein, liposomal doxorubicin and gold nanorods were incubated with RAW 264.7 macrophages and later injected intratumorally in breast tumors in immunocompetent mice. The combination of liposomal doxorubicin, with the hyperthermia produced upon irradiation of gold nanorods, resulted in greater tumor reduction respect to the same nanotherapeutics delivered without the aid of macrophages.

Because M1-like TAMs have no means to recognize antigens on cancer cells, or to phagocyte non-opsonized cancer cells, simply depleting M2-like TAMs or converting them to M1-like might not be sufficient to obtain a notable response or tumor eradication. In this direction, a recent development, *i.e.* Chimeric Antigen Receptor-Macrophage (CAR-M) therapy, has shown exciting results and is worth mentioning in this section. In CAR-M, autologous monocytes are differentiated to M1-like macrophages *ex vivo* and transduced with an adenovirus-encoded CAR transgene. With this strategy, developed by Klichinsky et al. in 2016 (100), CAR-modified macrophages eliminated tumor cells more effectively than non-CAR-modified M1-like macrophages *in vitro* and *in vivo* (101). The creators of this technology have developed the company CARISMA therapeutics which has an ongoing phase I clinical trial on CAR-M for treating HER2- overexpressing solid tumors (including squamous carcinoma) (NCT04660929). Likely, the success of CAR-M over CAR-T in solid tumors is related to the unique ability of macrophages to extravasate and penetrate stroma-rich solid tumors.

## Conclusions and Outlook

The literature supports the idea that TAMs in OSCC deserve high attention given the usefulness of knowing TAM density and phenotype for prognosis. The M2-like TAM marker CD206 better correlates with malignancy and progression, particularly to EGF-associated progression, while CD163 has also been found high in premalignant lesions.

The CD206+ M2-like TAMs/OSCC cell interaction could represent a marker of metastasis and malignancy in OSCC, just as the TMEM (tumor microenvironment of metastasis) has shown to be a relevant marker for breast cancer (102).

To target M2-like TAMs for drug delivery purposes, we believe that small molecular weight ligands of moderate affinity might have an edge over antibodies (*α*-CD206, *α*-CD163, and *α*-CSF1R) or nanobodies because of high stromal content and the binding site barrier.

Surprisingly, at least to our knowledge, therapeutic approaches aimed at depleting or reprogramming M2-like TAMs have not been reported in OSCC. In the future, therapeutic interventions on M2-like TAMs will likely be complemented with approaches to boost the anti-tumor response, such as check point inhibitor immunotherapy.

## Author Contributions

All authors contributed to the conception, writing, and editing of the manuscript. All authors contributed to the article and approved the submitted version.

## Funding

PS acknowledges support by the Estonian Research Council (grant: PUT PSG38 to PS) and a development fund of Tartu University.

## Conflict of Interest

The authors declare that the research was conducted in the absence of any commercial or financial relationships that could be construed as a potential conflict of interest.

## References

[1] Petersen PE. Oral Cancer Prevention and Control - The Approach of the World Health Organization. Oral Oncol (2009) 45:454–60. 10.1016/j.oraloncology.2008.05.023 18804412

[2] Markwell SM Weed SA. Tumor and Stromal-Based Contributions to Head and Neck Squamous Cell Carcinoma Invasion. Cancers (Basel) (2015) 7:382–406. 10.3390/cancers7010382 25734659 PMC4381264

[3] Varsha B Radhika M Makarla S Kuriakose M Satya Kiran GVV Padmalatha GV. Perineural Invasion in Oral Squamous Cell Carcinoma: Case Series and Review of Literature. J Oral Maxillofac Pathol (2015) 19:335–41. 10.4103/0973-029X.174630 PMC4774287 26980962

[4] Rani P Gupta A Mehrol C Singh M Khurana N Passey J. Clinicopathological Correlation of Tumor-Stroma Ratio and Inflammatory Cell Infiltrate With Tumor Grade and Lymph Node Metastasis in Squamous Cell Carcinoma of Buccal Mucosa and Tongue in 41 Cases With Review of Literature. J Cancer Res Ther (2020) 16:445–51. 10.4103/0973-1482.193113 32719249

[5] Pennacchiotti G Valdés-Gutiérrez F Alejandro González-Arriagada W Montes HF Maria J Parra R. SPINK7 Expression Changes Accompanied by HER2, P53 and RB1 can be Relevant in Predicting Oral Squamous Cell Carcinoma At a Molecular Level. Sci Rep 11:6939. 10.1038/s41598-021-86208-z PMC7994578 33767253

[6] Hanahan D Weinberg RA. Hallmarks of Cancer: The Next Generation. Cell (2011) 144:646–74. 10.1016/j.cell.2011.02.013 21376230

[7] Sonnenschein C Soto AM. The Aging of the 2000 and 2011 Hallmarks of Cancer Reviews: A Critique. J Biosci (2013) 38:651–63. 10.1007/s12038-013-9335-6 PMC3882065 23938395

[8] Bruna F Plaza A Arango M Espinoza I Conget P. Systemically Administered Allogeneic Mesenchymal Stem Cells do Not Aggravate the Progression of Precancerous Lesions: A New Biosafety Insight. Stem Cell Res Ther (2018) 9. 10.1186/s13287-018-0878-1 PMC5948822 29751770

[9] Bruna F Arango-Rodríguez M Plaza A Espinoza I Conget P. The Administration of Multipotent Stromal Cells At Precancerous Stage Precludes Tumor Growth and Epithelial Dedifferentiation of Oral Squamous Cell Carcinoma. Stem Cell Res (2017) 18:5–13. 10.1016/j.scr.2016.11.016 27939557

[10] Christopher AF Gupta M Bansal P. Micronome Revealed miR-19a/b as Key Regulator of SOCS3 During Cancer Related Inflammation of Oral Squamous Cell Carcinoma. Gene (2016) 594:30–40. 10.1016/j.gene.2016.08.044 27581787

[11] Singh PK Chandra G Bogra J Gupta R Kumar V Jain A. Association of Interleukin-6 Genetic Polymorphisms With Risk of OSCC in Indian Population. Meta Gene (2015) 4:142–51. 10.1016/j.mgene.2015.03.002 PMC4436510 26005639

[12] Liu R Li J Xie K Zhang T Lei Y Chen Y. FGFR4 Promotes Stroma-Induced Epithelial-to-Mesenchymal Transition in Colorectal Cancer. Cancer Res (2013) 73:5926–35. 10.1158/0008-5472.CAN-12-4718 23943801

[13] Colotta F Allavena P Sica A Garlanda C Mantovani A. Cancer-Related Inflammation, the Seventh Hallmark of Cancer: Links to Genetic Instability. Carcinogenesis (2009) 30:1073–81. 10.1093/carcin/bgp127 19468060

[14] Feller LL Khammissa RRAG Kramer BB Lemmer JJ. Oral Squamous Cell Carcinoma in Relation to Field Precancerisation: Pathobiology. Cancer Cell Int (2013) 13. 10.1186/1475-2867-13-31 PMC3626548 23552362

[15] Weber M Moebius P Büttner-Herold M Amann K Preidl R Neukam FW. Macrophage Polarisation Changes Within the Time Between Diagnostic Biopsy and Tumour Resection in Oral Squamous Cell Carcinomas-an Immunohistochemical Study. Br J Cancer (2015) 113:510–9. 10.1038/bjc.2015.212 PMC4522624 26110975

[16] Sica A Schioppa T Mantovani A Allavena P. Tumour-Associated Macrophages are a Distinct M2 Polarised Population Promoting Tumour Progression: Potential Targets of Anti-Cancer Therapy. Eur J Cancer (2006) 42:717–27. 10.1016/j.ejca.2006.01.003 16520032

[17] Gordon S Taylor PR. Monocyte and Macrophage Heterogeneity. Nat Rev Immunol (2005) 5:953–64. 10.1038/nri1733 16322748

[18] McWhorter FY Davis CT Liu WF. Physical and Mechanical Regulation of Macrophage Phenotype and Function. Cell Mol Life Sci (2015) 72:1303–16. 10.1007/s00018-014-1796-8 PMC4795453 25504084

[19] Zhang B Yao G Zhang Y Gao J Yang B Rao Z. M2-Polarized Tumor-Associated Macrophages are Associated With Poor Prognoses Resulting From Accelerated Lymphangiogenesis in Lung Adenocarcinoma. Clinics (2011) 66:1879–86. 10.1590/S1807-59322011001100006 PMC3203959 22086517

[20] Lapenna A De Palma M Lewis CE. Perivascular Macrophages in Health and Disease. Nat Rev Immunol (2018) 18:689–702. 10.1038/s41577-018-0056-9 30127389

[21] Melton DW McManus LM Gelfond JAL Shireman PK. Temporal Phenotypic Features Distinguish Polarized Macrophages In Vitro. Autoimmunity (2015) 48:161–76. 10.3109/08916934.2015.1027816 PMC4681525 25826285

[22] Mosser DM Edwards JP. Exploring the Full Spectrum of Macrophage Activation. Nat Rev Immunol (2008) 8:958–69. 10.1038/nri2448 PMC2724991 19029990

[23] Gordon S. Alternative Activation of Macrophages. Nat Rev Immunol (2003) 3:23–35. 10.1038/nri978 12511873

[24] Mantovani A Sozzani S Locati M Allavena P Sica A. Macrophage Polarization: Tumor-associated Macrophages as a Paradigm for Polarized M2 Mononuclear Phagocytes. Trends Immunol (2002) 23:549–55. 10.1016/S1471-4906(02)02302-5 12401408

[25] Barros MHM Hauck F Dreyer JH Kempkes B Niedobitek G. Macrophage Polarisation: An Immunohistochemical Approach for Identifying M1 and M2 Macrophages. PloS One (2013) 8. 10.1371/journal.pone.0080908 PMC3829941 24260507

[26] Wang N Liang H Zen K. Molecular Mechanisms That Influence the Macrophage M1-M2 Polarization Balance. Front Immunol (2014) 5. 10.3389/fimmu.2014.00614 PMC4246889 25506346

[27] Martinez FO Gordon S Locati M Mantovani A. Transcriptional Profiling of the Human Monocyte-to-Macrophage Differentiation and Polarization: New Molecules and Patterns of Gene Expression. J Immunol (2006) 177:7303–11. 10.4049/jimmunol.177.10.7303 17082649

[28] Petruzzi MNMR Cherubini K Salum FG de Figueiredo MAZ. Role of Tumour-Associated Macrophages in Oral Squamous Cells Carcinoma Progression: An Update on Current Knowledge. Diagn Pathol (2017) 12. 10.1186/s13000-017-0623-6 PMC5382416 28381274

[29] Mori K Hiroi M Shimada J Ohmori Y. Infiltration of M2 Tumor-Associated Macrophages in Oral Squamous Cell Carcinoma Correlates With Tumor Malignancy. Cancers (Basel) (2011) 3:3726–739. 10.3390/cancers3043726 PMC3763393 24213108

[30] Komohara Y Jinushi M Takeya M. Clinical Significance of Macrophage Heterogeneity in Human Malignant Tumors. Cancer Sci (2014) 105:1–8. 10.1111/cas.12314 24168081 PMC4317877

[31] Fabriek BO Dijkstra CD van den Berg TK. The Macrophage Scavenger Receptor CD163. Immunobiology (2005). 10.1016/j.imbio.2005.05.010 16164022

[32] Baay M Brouwer A Pauwels P Peeters M Lardon F. Tumor Cells and Tumor-Associated Macrophages: Secreted Proteins as Potential Targets for Therapy. Clin Dev Immunol (2011) 2011. 10.1155/2011/565187 PMC3227419 22162712

[33] Choi YK Fallert Junecko BA Klamar CR Reinhart TA. Characterization of Cells Expressing Lymphatic Marker LYVE-1 in Macaque Large Intestine During Simian Immunodeficiency Virus Infection Identifies a Large Population of Nonvascular LYVE-1+/DC-SIGN+ Cells. Lymphat Res Biol (2013) 11:26–34. 10.1089/lrb.2012.0019 23531182 PMC3609640

[34] Magnusson S Berg T. Extremely Rapid Endocytosis Mediated by the Mannose Receptor of Sinusoidal Endothelial Rat Liver Cells. Biochem J (1989) 257:651–6. 10.1042/bj2570651 PMC1135637 2930475

[35] Malovic I Sørensen KK Elvevold KH Nedredal GI Paulsen S Erofeev AV. The Mannose Receptor on Murine Liver Sinusoidal Endothelial Cells is the Main Denatured Collagen Clearance Receptor. Hepatology (2007) 45:1454–61. 10.1002/hep.21639 17518370

[36] Wang S Liu R Yu Q Dong L Bi Y Liu G. Metabolic Reprogramming of Macrophages During Infections and Cancer. Cancer Lett (2019) 452:14–22. 10.1016/j.canlet.2019.03.015 30905817

[37] Suárez-Sánchez FJ Lequerica-Fernández P Suárez-Canto J Rodrigo JP Rodriguez-Santamarta T Domínguez-Iglesias F. Macrophages in Oral Carcinomas: Relationship With Cancer Stem Cell Markers and PD-L1 Expression. Cancers (Basel) (2020) 12:1–14. 10.3390/cancers12071764 PMC7408350 32630659

[38] Takeya M Komohara Y. Role of Tumor-Associated Macrophages in Human Malignancies: Friend or Foe? Pathol Int (2016) 66:491–505. 10.1111/pin.12440 27444136

[39] Weber M Wehrhan F Baran C Agaimy A Büttner-Herold M Öztürk H. Malignant Transformation of Oral Leukoplakia is Associated With Macrophage Polarization. J Transl Med (2020) 18. 10.1186/s12967-019-02191-0 PMC6945578 31910881

[40] Chiu KC Lee CH Liu SY Chou YT Huang RY Huang SM. Polarization of Tumor-Associated Macrophages and Gas6/Axl Signaling in Oral Squamous Cell Carcinoma. Oral Oncol (2015) 51:683–9. 10.1016/j.oraloncology.2015.04.004 25910588

[41] Wehrhan F Büttner-Herold M Hyckel P Moebius P Preidl R Distel L. Increased Malignancy of Oral Squamous Cell Carcinomas (Oscc) is Associated With Macrophage Polarization in Regional Lymph Nodes - an Immunohistochemical Study. BMC Cancer (2014) 14. 10.1186/1471-2407-14-522 PMC4223559 25042135

[42] Mori K Haraguchi S Hiori M Shimada J Ohmori Y. Tumor-Associated Macrophages in Oral Premalignant Lesions Coexpress CD163 and STAT1 in a Th1-dominated Microenvironment. BMC Cancer (2015) 15. 10.1186/s12885-015-1587-0 PMC4525742 26242181

[43] Metwaly H Maruyama S Yamazaki M Tsuneki M Abé T Jen KY. Parenchymal-Stromal Switching for Extracellular Matrix Production on Invasion of Oral Squamous Cell Carcinoma. Hum Pathol (2012) 43:1973–81. 10.1016/j.humpath.2012.02.006 22575259

[44] Weber M Iliopoulos C Moebius P Büttner-Herold M Amann K Ries J. Prognostic Significance of Macrophage Polarization in Early Stage Oral Squamous Cell Carcinomas. Oral Oncol (2016) 52:75–84. 10.1016/j.oraloncology.2015.11.001 26728105

[45] Shigeoka M Koma Y Kodama T Nishio M Akashi M Yokozaki H. Intraepithelial CD163+ Macrophages in Tongue Leukoplakia Biopsy: A Promising Tool for Cancer Screening. Oral Dis (2020) 26:527–36. 10.1111/odi.13269 31886947

[46] Patankar SR Wankhedkar DP Tripathi NS Bhatia SN Sridharan G. Extracellular Matrix in Oral Squamous Cell Carcinoma: Friend or Foe? Indian J Dent Res (2016) 27:184–9. 10.4103/0970-9290.183125 27237211

[47] Polz-Dacewicz M Strycharz-Dudziak M Dworzański J Stec A Kocot J. Salivary and Serum IL-10, Tnf-α, Tgf-β, VEGF Levels in Oropharyngeal Squamous Cell Carcinoma and Correlation With HPV and EBV Infections. Infect Agent Cancer (2016) 11. 10.1186/s13027-016-0093-6 PMC4992298 27547238

[48] Wang S Sun M Gu C Wang X Chen D Zhao E. Expression of CD163, interleukin-10, and Interferon-Gamma in Oral Squamous Cell Carcinoma: Mutual Relationships and Prognostic Implications. Eur J Oral Sci (2014) 122:202–9. 10.1111/eos.12131 24796206

[49] Kumar V Gabrilovich DI. Hypoxia-Inducible Factors in Regulation of Immune Responses in Tumour Microenvironment. Immunology (2014) 143:512–9. 10.1111/imm.12380 PMC4253499 25196648

[50] Chung FT Lee KY Wang CW Heh CC Chan YF Chen HW. Tumor-Associated Macrophages Correlate With Response to Epidermal Growth Factor Receptor-Tyrosine Kinase Inhibitors in Advanced non-Small Cell Lung Cancer. Int J Cancer (2012) 131. 10.1002/ijc.27403 22174092

[51] Shigeoka M Urakawa N Nishio M Takase N Utsunomiya S Akiyama H. Cyr61 Promotes CD204 Expression and the Migration of Macrophages Via MEK/ERK Pathway in Esophageal Squamous Cell Carcinoma. Cancer Med (2015) 4:437–46. 10.1002/cam4.401 PMC4380969 25620088

[52] Jayasingam SD Citartan M Thang TH Mat Zin AA Ang KC Ch’ng ES. Evaluating the Polarization of Tumor-Associated Macrophages Into M1 and M2 Phenotypes in Human Cancer Tissue: Technicalities and Challenges in Routine Clinical Practice. Front Oncol (2020) 9. 10.3389/fonc.2019.01512 PMC6992653 32039007

[53] Haque ASMR Moriyama M Kubota K Ishiguro N Sakamoto M Chinju A. CD206+ Tumor-Associated Macrophages Promote Proliferation and Invasion in Oral Squamous Cell Carcinoma Via EGF Production. Sci Rep (2019) 9. 10.1038/s41598-019-51149-1 PMC6787225 31601953

[54] Fujii N Shomori K Shiomi T Nakabayashi M Takeda C Ryoke K. Cancer-Associated Fibroblasts and CD163-positive Macrophages in Oral Squamous Cell Carcinoma: Their Clinicopathological and Prognostic Significance. J Oral Pathol Med (2012) 41:444–51. 10.1111/j.1600-0714.2012.01127.x 22296275

[55] Lopez-Yrigoyen M Cassetta L Pollard JW. Macrophage Targeting in Cancer. Ann N Y Acad Sci (2020) 14–24. 10.1111/nyas.14377 32445205

[56] Peranzoni E Lemoine J Vimeux L Feuillet V Barrin S Kantari-Mimoun C. Macrophages Impede CD8 T Cells From Reaching Tumor Cells and Limit the Efficacy of Anti–PD-1 Treatment. Proc Natl Acad Sci (2018) 115:E4041–50. 10.1073/pnas.1720948115 PMC5924916 29632196

[57] Pathria P Louis TL Varner JA. Targeting Tumor-Associated Macrophages in Cancer. Trends Immunol (2019) 40:310–27. 10.1016/j.it.2019.02.003 30890304

[58] Wies Mancini VSB Pasquini JM Correale JD Pasquini LA. Microglial Modulation Through Colony-Stimulating Factor-1 Receptor Inhibition Attenuates Demyelination. Glia (2019) 67:291–308. 10.1002/glia.23540 30456797

[59] El Khoury J Toft M Hickman SE Means TK Terada K Geula C. Ccr2 Deficiency Impairs Microglial Accumulation and Accelerates Progression of Alzheimer-like Disease. Nat Med (2007) 13:432–38. 10.1038/nm1555 17351623

[60] Ponomarev ED Shriver LP Dittel BN. Cd40 Expression by Microglial Cells Is Required for Their Completion of a Two-Step Activation Process During Central Nervous System Autoimmune Inflammation. J Immunol (2014) 176:1402–10. 10.4049/jimmunol.176.3.1402 16424167

[61] Lei F Cui N Zhou C Chodosh J Vavvas DG Paschalis EI. CSF1R Inhibition by a Small-Molecule Inhibitor is Not Microglia Specific; Affecting Hematopoiesis and the Function of Macrophages. Proc Natl Acad Sci U S A (2020) 117:23336–8. 10.1073/pnas.1922788117 PMC7519218 32900927

[62] Katzenelenbogen Y Sheban F Yalin A Yofe I Svetlichnyy D Jaitin DA. Coupled scRNA-Seq and Intracellular Protein Activity Reveal an Immunosuppressive Role of TREM2 in Cancer. Cell (2020) 182:872–85. 10.1016/j.cell.2020.06.032 32783915

[63] Etzerodt A Tsalkitzi K Maniecki M Damsky W Delfini M Baudoin E. Specific Targeting of CD163+ Tams Mobilizes Inflammatory Monocytes and Promotes T Cell-Mediated Tumor Regression. J Exp Med (2019) 216:2394–411. 10.1084/jem.20182124 PMC6781002 31375534

[64] Kitamura T Doughty-Shenton D Cassetta L Fragkogianni S Brownlie D Kato Y. Monocytes Differentiate to Immune Suppressive Precursors of Metastasis-Associated Macrophages in Mouse Models of Metastatic Breast Cancer. Front Immunol (2018) 8:1–14. 10.3389/fimmu.2017.02004 PMC5776392 29387063

[65] Sato J van Osdol W Weinstein JN Perez-Bacete MJ. Micropharmacology of Monoclonal Antibodies in Solid Tumors: Direct Experimental Evidence for a Binding Site Barrier. Cancer Res (1992) 52:5144–53. 1327501

[66] Lu G Fakurnejad S Martin BA van den Berg NS van Keulen S Nishio N. Predicting Therapeutic Antibody Delivery Into Human Head and Neck Cancers. Clin Cancer Res (2020) 26:2582–94. 10.1158/1078-0432.CCR-19-3717 PMC9398035 31980465

[67] Lu G Nishio N van den Berg NS Martin BA Fakurnejad S van Keulen S. Co-Administered Antibody Improves Penetration of Antibody–Dye Conjugate Into Human Cancers With Implications for Antibody–Drug Conjugates. Nat Commun (2020) 11:1–11. 10.1038/s41467-020-19498-y 33168818 PMC7652891

[68] Movahedi K Schoonooghe S Laoui D Houbracken I Waelput W Breckpot K. Nanobody-Based Targeting of the Macrophage Mannose Receptor for Effective In Vivo Imaging of Tumor-Associated Macrophages. Cancer Res (2012) 72:4165–77. 10.1158/0008-5472.CAN-11-2994 22719068

[69] Azad AK Rajaram MVS Metz WL Cope FO Blue MS Vera DR. -Tilmanocept, a New Radiopharmaceutical Tracer for Cancer Sentinel Lymph Nodes, Binds to the Mannose Receptor (Cd206). J Immunol (2015) 195:2019–29. 10.4049/jimmunol.1402005 PMC4543904 26202986

[70] Jameson B Baribaud F Pöhlmann S Ghavimi D Mortari F Doms RW. Expression of DC-SIGN by Dendritic Cells of Intestinal and Genital Mucosae in Humans and Rhesus Macaques. J Virol (2002) 76:1866–75. 10.1128/JVI.76.4.1866-1875.2002 PMC135921 11799181

[71] Zhang F Parayath NN Ene CI Stephan SB Koehne AL Coon ME. Genetic Programming of Macrophages to Perform Anti-Tumor Functions Using Targeted mRNA Nanocarriers. Nat Commun (2019) 10. 10.1038/s41467-019-11911-5 PMC6722139 31481662

[72] Jaynes JM Sable R Ronzetti M Bautista W Knotts Z Abisoye-Ogunniyan A. Mannose Receptor (CD206) Activation in Tumor-Associated Macrophages Enhances Adaptive and Innate Antitumor Immune Responses. Sci Transl Med (2020) 12. 10.1126/scitranslmed.aax6337 PMC7832040 32051227

[73] Jaynes JM Lopez HW Martin GR Yates C Garvin CE. Peptides having anti-inflammatory properties. Patent (2015) .

[74] Simón-Gracia L Scodeller P Fuentes SS Vallejo VG Ríos X San Sebastián E. Application of Polymersomes Engineered to Target p32 Protein for Detection of Small Breast Tumors in Mice. Oncotarget (2018) 9:18682–97. 10.18632/oncotarget.24588 PMC5922347 29721153

[75] Simón-Gracia L Hunt H Teesalu T. Peritoneal Carcinomatosis Targeting With Tumor Homing Peptides. Molecules (2018) 23. 10.3390/molecules23051190 PMC6100015 29772690

[76] Simon-Gracia L Hunt H Scodeller P Gaitzsch J Kotamraju VR Sugahara KN. iRGD Peptide Conjugation Potentiates Intraperitoneal Tumor Delivery of Paclitaxel With Polymersomes. Biomaterials (2016) 104:247–57. 10.1016/j.biomaterials.2016.07.023 PMC5687559 27472162

[77] Wonder E Simón-Gracia L Scodeller P Majzoub RN Kotamraju VR Ewert KK. Competition of Charge-Mediated and Specific Binding by Peptide-Tagged Cationic Liposome–DNA Nanoparticles In Vitro and In Vivo. Biomaterials (2018) 166:52–63. 10.1016/j.biomaterials.2018.02.052 29544111 PMC5944340

[78] Diaz Bessone MI Simón-Gracia L Scodeller P Ramirez MDLA Lago Huvelle MA Soler-Illia GJAA. IRGD-guided tamoxifen polymersomes inhibit estrogen receptor transcriptional activity and decrease the number of breast cancer cells with self-renewing capacity. J Nanobiotechnology (2019) 17. 10.1186/s12951-019-0553-4 PMC6898937 31812165

[79] Ikemoto H Lingasamy P Willmore AMA Hunt H Kurm K Tammik O. Hyaluronan-Binding Peptide for Targeting Peritoneal Carcinomatosis. Tumor Biol (2017) 39. 10.1177/1010428317701628 PMC5697747 28468593

[80] Scodeller P Asciutto EK. Targeting Tumors Using Peptides. Mol (2020) 25:808. 10.3390/molecules25040808 PMC7070747 32069856

[81] Mann AP Scodeller P Hussain S Braun GB Mölder T Toome K. Identification of a Peptide Recognizing Cerebrovascular Changes in Mouse Models of Alzheimer’s Disease. Nat Commun (2017) 8:1–11. 10.1038/s41467-017-01096-0 29123083 PMC5680235

[82] Cieslewicz M Tang J Yu JL Cao H Zavaljevski M Motoyama K. Targeted Delivery of Proapoptotic Peptides to Tumor-Associated Macrophages Improves Survival. Proc Natl Acad Sci U S A (2013) 110:15919–24. 10.1073/pnas.1312197110 PMC3791765 24046373

[83] Scodeller P Simón-Gracia L Kopanchuk S Tobi A Kilk K Säälik P. Precision Targeting of Tumor Macrophages With a CD206 Binding Peptide. Sci Rep (2017) 7:1–12. 10.1038/s41598-017-14709-x 29116108 PMC5676682

[84] Asciutto EK Kopanchuk S Lepland A Simón-Gracia L Aleman C Teesalu T. Phage-Display-Derived Peptide Binds to Human CD206 and Modeling Reveals a New Binding Site on the Receptor. J Phys Chem B (2019) 123:1973–82. 10.1021/acs.jpcb.8b11876 30768279

[85] Lepland A Asciutto EK Malfanti A Simón-Gracia L Sidorenko V Vicent MJ. Targeting Pro-Tumoral Macrophages in Early Primary and Metastatic Breast Tumors With CD206-binding mUNO Peptide. Mol Pharm (2020) 17:2518–31. 10.1021/acs.molpharmaceut.0c00226 32421341

[86] Figueiredo P Lepland A Scodeller P Fontana F Torrieri G Tiboni M. Peptide-Guided Resiquimod-Loaded Lignin Nanoparticles Convert Tumor-Associated Macrophages From M2 to M1 Phenotype for Enhanced Chemotherapy. Acta Biomater (2020). 10.1016/j.actbio.2020.09.038 33011297

[87] Savage P Horton V Moore J Owens M Witt P Gore ME. A Phase I Clinical Trial of Imiquimod, an Oral Interferon Inducer, Administered Daily. Br J Cancer (1996) 74:1482–6. 10.1038/bjc.1996.569 PMC2074776 8912549

[88] Pockros PJ Guyader D Patton H Tong MJ Wright T McHutchison JG. Oral Resiquimod in Chronic HCV Infection: Safety and Efficacy in 2 Placebo-Controlled, Double-Blind Phase IIa Studies. J Hepatol (2007) 47:174–82. 10.1016/j.jhep.2007.02.025 17532523

[89] Puig-Kröger A Sierra-Filardi E Domínguez-Soto A Samaniego R Corcuera MT Gómez-Aguado F. Folate Receptor β is Expressed by Tumor-Associated Macrophages and Constitutes a Marker for M2 Anti-Inflammatory/Regulatory Macrophages. Cancer Res (2009) 69:9395–403. 10.1158/0008-5472.CAN-09-2050 19951991

[90] Zhang F Ayaub EA Wang B Puchulu-Campanella E Li Y Hettiarachchi SU. Reprogramming of Profibrotic Macrophages for Treatment of Bleomycin-Induced Pulmonary Fibrosis. EMBO Mol Med (2020) 12. 10.15252/emmm.202012034 PMC7411553 32597014

[91] Vinnakota K Zhang Y Selvanesan BC Topi G Salim T Sand-Dejmek J. M2-Like Macrophages Induce Colon Cancer Cell Invasion Via Matrix Metalloproteinases. J Cell Physiol (2017) 232:3468–80. 10.1002/jcp.25808 28098359

[92] Lolmede K Campana L Vezzoli M Bosurgi L Tonlorenzi R Clementi E. Inflammatory and Alternatively Activated Human Macrophages Attract Vessel-Associated Stem Cells, Relying on Separate HMGB1- and MMP-9-dependent Pathways. J Leukoc Biol (2009) 85:779–87. 10.1189/jlb.0908579 19197071

[93] Miller MA Zheng YR Gadde S Pfirschke C Zope H Engblom C. Tumour-Associated Macrophages Act as a Slow-Release Reservoir of Nano-Therapeutic Pt(IV) Pro-Drug. . Nat Commun (2015) 6. 10.1038/ncomms9692 PMC4711745 26503691

[94] Wyatt Shields C Evans MA Wang LLW Baugh N Iyer S Wu D. Cellular Backpacks for Macrophage Immunotherapy. Sci Adv (2020) 6. 10.1126/sciadv.aaz6579 PMC7190308 32494680

[95] Champion JA Mitragotri S. Role of Target Geometry in Phagocytosis. Proc Natl Acad Sci U S A (2006) 103:4930–34. 10.1073/pnas.0600997103 PMC1458772 16549762

[96] Torrieri G Fontana F Figueiredo P Liu Z Ferreira MPA Talman V. Dual-Peptide Functionalized Acetalated Dextran-Based Nanoparticles for Sequential Targeting of Macrophages During Myocardial Infarction. Nanoscale (2020) 12:2350–8. 10.1039/c9nr09934d 31930241

[97] Fogal V Zhang L Krajewski S Ruoslahti E. Mitochondrial/Cell-Surface Protein P32/gC1qR as a Molecular Target in Tumor Cells and Tumor Stroma. Cancer Res (2008) 68:7210–8. 10.1158/0008-5472.CAN-07-6752 PMC2562323 18757437

[98] Simón-Gracia L Scodeller P Fuentes SS Vallejo VG Ríos X Sebastián ES. Application of Polymersomes Engineered to Target p32 Protein for Detection of Small Breast Tumors in Mice. Oncotarget (2018) 9:18682–97. 10.18632/oncotarget.24588 PMC5922347 29721153

[99] Nguyen V Min HK Kim DH Kim CS Han J Park JO. Macrophage-Mediated Delivery of Multifunctional Nanotherapeutics for Synergistic Chemo-Photothermal Therapy of Solid Tumors. ACS Appl Mater Interfaces (2020) 12:10130–41. 10.1021/acsami.9b23632 32041404

[100] Anderson NR Minutolo NG Gill S Klichinsky M. Macrophage-Based Approaches for Cancer Immunotherapy. Cancer Res (2020) 81:1201–8. 10.1158/0008-5472.can-20-2990 33203697

[101] Klichinsky M Ruella M Shestova O Lu XM Best A Zeeman M. Human Chimeric Antigen Receptor Macrophages for Cancer Immunotherapy. Nat Biotechnol (2020) 38:947–53. 10.1038/s41587-020-0462-y PMC7883632 32361713

[102] Karagiannis GS Pastoriza JM Wang Y Harney AS Entenberg D Pignatelli J. Neoadjuvant Chemotherapy Induces Breast Cancer Metastasis Through a TMEM-mediated Mechanism. Sci Transl Med (2017) 9:1–15. 10.1126/scitranslmed.aan0026 PMC5592784 28679654

